# Beneficial Effects of Proanthocyanidins in the Cardiac Alterations Induced by Aldosterone in Rat Heart through Mineralocorticoid Receptor Blockade

**DOI:** 10.1371/journal.pone.0111104

**Published:** 2014-10-29

**Authors:** Beatriz Martín-Fernández, Natalia de las Heras, María Valero-Muñoz, Sandra Ballesteros, Yi-Zhou Yao, Peter G. Stanton, Peter J. Fuller, Vicente Lahera

**Affiliations:** 1 Department of Physiology, School of Medicine, Universidad Complutense, Madrid, Spain; 2 Prince Henry’s Institute of Medical Research, Clayton, Victoria, Australia; Universidad de La Laguna, Spain

## Abstract

Aldosterone administration in rats results in several cardiac alterations. Previous studies have demonstrated that proanthocyanidins, phenolic bioactive compounds, have cardioprotective effects. We studied the potential beneficial effects of the proanthocyanidin-rich almond skin extract (PASE) on the cardiac alterations induced by aldosterone-salt treatment, their effects in mineralocorticoid receptor activity and we sought to confirm proanthocyanidins as the specific component of the extract involved in the beneficial cardiac effects. Male Wistar rats received aldosterone (1 mg/Kg/day) +1% NaCl for 3 weeks. Half of the animals in each group were simultaneously treated with either PASE (100 mg/Kg/day) or spironolactone (200 mg/Kg/day). The ability of PASE to act as an antagonist of the mineralocorticoid receptor was examined using a transactivation assay. High performance liquid chromatography was used to identify and to isolate proanthocyanidins. Hypertension and diastolic dysfunction induced by aldosterone were abolished by treatment with PASE. Expression of the aldosterone mediator SGK-1, together with fibrotic, inflammatory and oxidative mediators were increased by aldosterone-salt treatment; these were reduced by PASE. Aldosterone-salt induced transcriptional activity of the mineralocorticoid receptor was reduced by PASE. HPLC confirmed proanthocyanidins as the compound responsible for the beneficial effects of PASE. The effects of PASE were comparable to those seen with the mineralocorticoid antagonist, spironolactone. The observed responses in the aldosterone-salt treated rats together with the antagonism of transactivation at the mineralocorticoid receptor by PASE provides evidence that the beneficial effect of this proanthocyanidin-rich almond skin extract is via as a mineralocorticoid receptor antagonist with proanthocyanidins identified as the compounds responsible for the beneficial effects of PASE.

## Background

Aldosterone exerts direct cardiac effects that contribute to pathological structural remodeling in the ventricular myocardium [1], [2]. In the clinical setting, elevated serum levels of aldosterone have been shown to be associated with high mortality in patients with severe congestive heart failure [3] and several clinical trials have established the beneficial effects of mineralocorticoid receptor antagonists for the treatment of heart failure [4], [5]. It has been demonstrated that aldosterone promotes myocardial perivascular and interstitial fibrosis [6], [7] and left ventricular hypertrophy [8]. Moreover, the flexibility of myocardial tissue is reduced, increasing the filling pressure of the heart and contributing to diastolic dysfunction [9]. On the other hand, the presence of cardiac hypertrophy is known to be an important risk factor for cardiovascular morbidity and mortality [10]–[12].

Proanthocyanidins are a large family of phenolic bioactive compounds (oligomers or polymers) composed of flavan-3-ol monomer subunits linked together. Based on their composition they are subdivided into three different families: procyanidins -composed exclusively of catechin and epicatechin monomers; prodelphinidins –containing at least one unit of gallocatechin or epigallocatechin together with units of catechin or epicatechin; and properlagonidins- containing at least one unit of afzelechin or epiafzelechin together with units of catechin or epicatechin. Proanthocyanidins have shown antioxidant [13], anti-inflammatory [14], anti-hypertensive [15], anti-platelet [16], anti-thrombotic [17] and hypocholesterolemic [18] activities. Furthermore, epidemiological studies have strongly suggested that regular consumption of proanthocyanidins may decrease the risk of cardiovascular diseases [19]. Polyphenolic constituents including oligomeric proanthocyanidins and resveratrol in red wine have been demonstrated to have cardioprotective effects [20], [21]. Polyphenols’ health properties have been attributed to the direct antioxidant effect of these phytochemicals, which act as free radical scavengers. However, recent data has revealed that polyphenols could interact with cell signaling pathways and modulate the activity of transcription factors with consequent regulation of gene expression [22]–[24]. This suggests that these cellular and molecular targets mediate the most relevant mechanisms of action underlying the biological effects of polyphenols.

We [25], [26], and others [27]–[30] have previously characterized a model in which administration of aldosterone plus 1% salt to rats results in cardiac hypertrophy, fibrosis, hypertension and diastolic dysfunction. This response, which is mediated by the mineralocorticoid receptor (MR), is attenuated by the MR antagonists, spironolactone and eplerenone [30], [31]. Although both antagonists of the mineralocorticoid receptor in current clinical use, spironolactone and eplerenone, are steroidal compounds there are now several reports of non-steroidal compounds with potent mineralocorticoid-specificity-antagonist activity [32]–[34].

None of the previous studies has examined the effect of proanthocyanidins in a cardiac disease model induced by aldosterone where many adverse cardiovascular alterations including inflammation, fibrosis and oxidative stress encounter. Therefore, since previous studies have shown beneficial effects of proanthocyanidins on cardiovascular diseases, the purpose of this study was to evaluate the effects of a proanthocyanidin-rich extract obtained from almond skin on cardiac alterations induced by aldosterone-salt administration and to further investigate the molecular mechanisms involved. Here, we analyzed structural, functional and molecular alterations induced in the rat heart by mineralocorticoid-salt treatment as well as the effects of PASE (Proanthocyanidin-rich Almond Skin Extract) on the serum and glucocorticoid regulated kinase type 1 (SGK-1) gene expression, recognized transcriptional target of MR actions. In order to further study the mechanisms of the effects of PASE, we analyzed the aldosterone-induced transcriptional activity of the mineralocorticoid receptor in the presence of increasing doses of PASE. Furthermore, we sought to confirm that proanthocyanidins were the extract compounds involved in the cardiac effects so; we obtained purified fractions of PASE by High performance liquid chromatography and, analyzed the aldosterone-induced transcriptional activity of the mineralocorticoid receptor in the presence of the purified fractions. The effects of PASE alone were compared with those of spironolactone.

## Methods

### Experimental design and animals

Forty male Wistar rats (256±3 g; Harlan Ibérica, Barcelona, Spain) were used in the study according to the guidelines for ethical care of experimental animals of the European Union and granted and approved by the Universidad Complutense Ethics Review Board following the National Guideline 53/2013. The Universidad Complutense Ethics Review Board specifically approved this study. Rats were fed standard rat chow and tap water *ad libitum* and kept in a quiet room at constant temperature (20 to 22°C) and humidity (50 to 60%). A control group and 4 experimental groups of animals were used in the study, 8 animals each group. Before allocating animals to treatment, blood pressure was measured to group them under the same mean blood pressure by the tail-cuff method. Aldosterone (1 mg/Kg/day) dissolved in corn oil or vehicle alone was subcutaneously injected once daily for 3 weeks; these rats received NaCl 1% as drinking water. Half of the animals in each group were simultaneously treated with either PASE (100 mg/Kg/day in the drinking water) or spironolactone as a positive control (200 mg/Kg/day, subcutaneous). PASE (25.1% proanthocyanidins w/w), used in this study contains the phenolic fraction of almonds (*Prunus dulcis*) with different degrees of polymerization, and is obtained by a standardized process, which concentrates the procyanidins, properlagonidins and prodelphinidins from almonds, including oligomers and polymers [35]–[37]. At the end of the treatment period, animals were anesthetized (Ketamine, Imalgene 1000, 70 mg/Kg, and Xilacine, Rompun 2%, 6 mg/Kg; intraperitoneal injection) and a catheter (Science FT211B of 1.6F diameter; Ontario, Canada) was inserted and advanced through the right carotid artery getting into the left ventricle as previously described [38]. The catheter was connected to a data acquisition system (PowerLab/800. ADInstruments, Castle Hill, Australia). Signals were monitored and digitally stored for their analysis with the commercial software Chart for Windows v4.2 (London, United Kingdom). Systolic and diastolic blood pressure (SBP and DBP), heart rate (HR), left ventricle end diastolic pressure (LVEDP), left ventricle systolic pressure (LVSP), the first derivative of LV pressure rise over time (+dP/dt) and the first derivative of LV pressure rise over time (−dP/dt) were measured. After measuring hemodynamic parameters, the animals were sacrificed by exsanguinations through the catheter inserted in the carotid artery, the heart removed, weighed and rapidly frozen in liquid nitrogen for molecular studies. The ratio of heart to body weight was used as the index of cardiac hypertrophy.

### Cardiac Collagen Quantification

A piece of LV from each rat was fixed in 3, 7% paraformaldehyde for 24 hours, and then stored in 70% alcohol until its use. The tissues were dehydrated, and fixed in paraffin and then cut into 4 µm slices. These slices were stained with Sirius Red F3BA (0.5% in saturated aqueous picric acid; Aldrich Chemical Company, Madrid, Spain). Quantification of collagen content was performed using an image analysis system (Leica Microsystems, Barcelona, Spain). The analyses were performed in four different sections of each slide of the heart and ten photographs from each section were taken. A single investigator, unaware of the experimental groups, performed these analyses.

### Real Time RT-PCR to Detect mRNA Expression

#### RNA isolation

Frozen rat hearts were pulverized in liquid nitrogen. RNA isolation was performed using an RNA extraction kit (Qiagen Sciences, Maryland, USA) and quantified by measurement of optical density at 260 nm (BioPhotometer, Eppendorf, Hamburg, Germany). The RNAs were stored at –80°C until their use.

#### cDNA synthesis

Genomic DNA was eliminated from the DNA with a mixture of gDNA Wipeout Buffer and RNPASE free water incubated for 2 min at 42°C. Then, 1 µg of total RNA was reverse transcribed using Quantiscript Reverse Transcriptase for 15 min at 42°C and 3 min at 95°C (Qiagen, Sciences, Maryland, USA).

#### Quantitative RT-PCR analysis

Real-time PCR was performed using a SmartCycler (Cepheid, Sunnyvale, California, USA). Taqman technology was used for qRT-PCR and Taq DNA polymerase (Qiagen Sciences, Maryland, USA) was used. Oligonucleotides, modified with fluorescence label at the 5′-end and with quencher at the 3′-end, were added to a reaction system. The relative quantitation of the gene expression was performed using the comparative C_T_ method [39]. The data was normalized using 18 S ribosomal RNA and expressed as % relative expression vs. control group. Table 1 shows primers sequences.

**Table 1:** Primers sequences.

Genes	Sense	Antisense	Probe
18S	5′CGCAAATTACCCACTCCCGACCC3′	5′GGCTACCACATCCAAGGAAG3′	5′ CAATTACAGGGCCTCGAAAGA 3′
CTGF	5′TGGCCCTGACCCAACTATGAT3′	5′GCACTTTTTGCCCTTCTTAATGTT3′	5′AGGCCAACTGCCTGGTCCAGACCA 3′
TGF-β	5′GGGCTTTCGCTTCAGTGCT 3′	5′TCGGTTCATGTCATGGATGGT3′	5′TCAGTCCCAAACGTCGAGGTGACCTG3′
MMP-2	5′CGTGGTGAGATCTTCTTCTTCAAGGA3′	5′CCTCATACACAGCGTCAATCTTTTC3′	5′ACACCACGTGACAAGCCCACAGGTC 3′
TIMP-2	5′GGAGGAAAGAAGGAATATCTAATTGCAG3′	5′CCAGGGCACAATAAAGTCACAGA3′	5′CATCTTGCCATCTCCTTCCGCCTTCC3′
IL-1β	5′TCTTCGAGGCACAAGGCAC3′	5′CAGAGGTCCAGGTCCTGGAA3′	5′ACCTGAGCTCGCCAGTGAAATGATGGCTT3′
TNF-α	5′GGTGATCGGTCCCAACAAGGA 3′	5′CACGCTGGCTCAGCCACTC 3′	5′TGGCCCAGACCCTCACACTCAGATCA3′
eNOS	5′AAGACGCTGCTTGGGATCC3′	5′AGCCTGGGAACCACTCCTTT3′	5′AGGAAGTTACAGAGCCGGCCCACCC3′
p22phox	5′GGACAGAAGTACCTGACCGCT3′	5′CAGGCACGGACAGCAGTAAG3′	5′AGGACAGCCCGGACGTAGTAATTTCTGGT3′
SGK-1	5′GCACGCCTGAGTATCTCGC3′	5′AGGCCATAGAGCATCTCATACAAGAC3′	5′CCCGAGGCACCACCAGTCCACT3′

GeneBank accession numbers: 18S NR045132.1, CTGF AC127189.4, TGF-β NM021578.2, MMP-2 BC074013.1, TIMP-2 BC084714.1, IL-1β, NG008851.1, TNF-α NM012675.3, eNOS NM021838.2, p22phox NM024160.1 and SGK-1 NM001193569.1.

### Tissue culture and transactivation assay

The transactivation assays were performed in CV-1 cells as described previously [40]. The cells were seeded at a density of 8×10^4^ cells/well in 12-well plates in Dulbecco’s modified Eagle’s medium (DMEM; Sigma) +10% FBS and incubated overnight before transfection. Transfections were performed using FuGene6 (Roche Molecular Biochemicals, Indianapolis, IN) as per manufacturer’s protocol, and the medium was changed to DMEM supplemented with 10% charcoal-stripped FBS. The cells were transfected with 500 ng of expression vector containing full-length human mineralocorticoid receptor (hMR) together with 500 ng of the luciferase reporter plasmid MMTV-LUC. The hMR expression construct is pRShMR [41]. Post-transfection, the cells were incubated with spironolactone (100 nM) or PASE (10 nM, 100 nM and 1 µM) in the presence or absence of aldosterone (1 nM) for 24 h. The “n” per experiment was 4 and all in vitro experiments are representative of 3 independent experiments. Luciferase activity was determined using the Dual Luciferase Assay system (Promega, Madison, MI) according to the manufacturer’s instructions. The MMTV-Luc plasmid described previously [42] was used and pRL-tk plasmid containing the Renilla luciferase gene (Promega, Madison, MI) was used as a control. Light units were measured in an EnVision Multilabel Reader (PerkinElmer, Waltham, MA).

### High performance liquid chromatography (HPLC)

**Figure 1:**
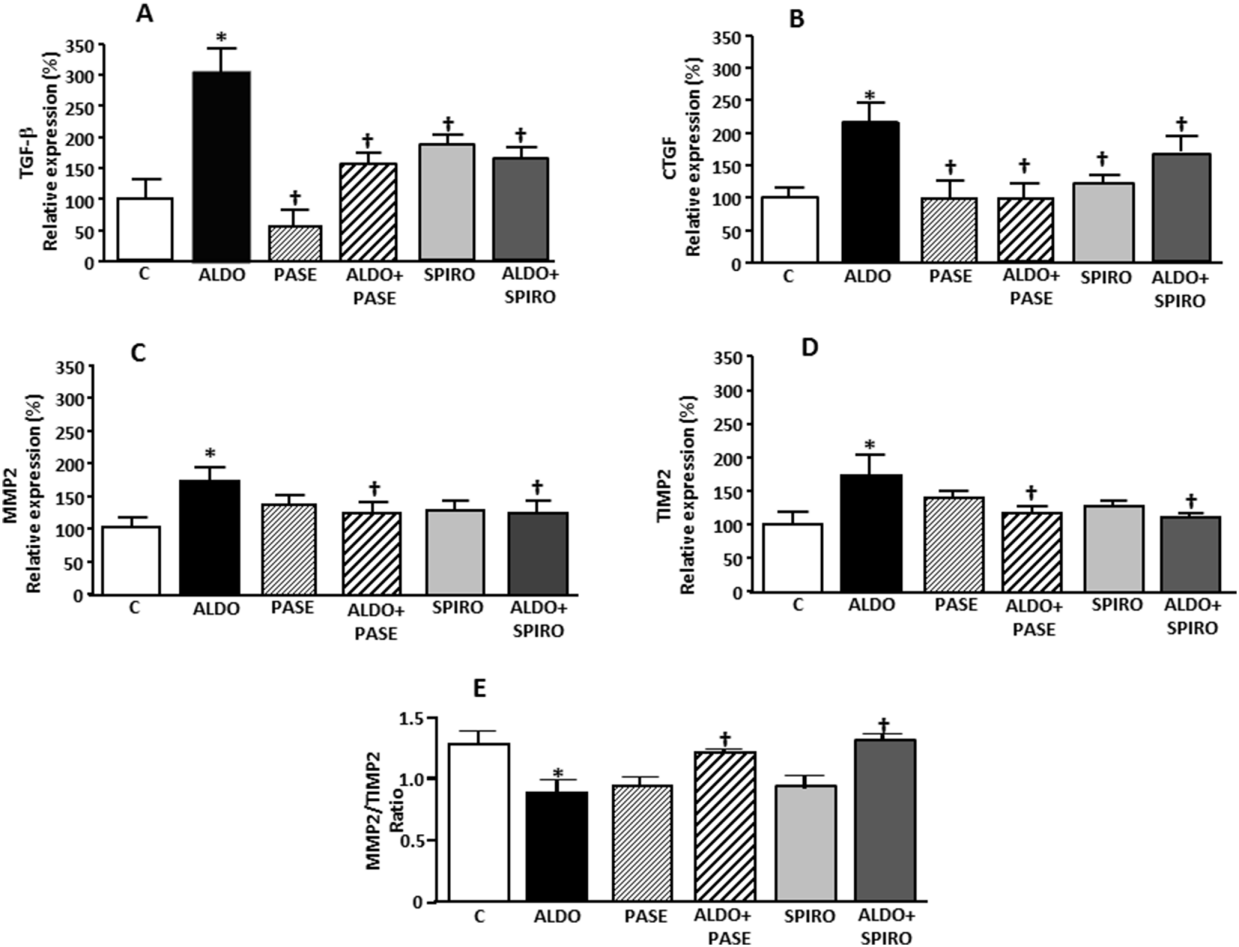
TGF-β (A), CTGF (B), MMP2 (C), TIMP2 (D) mRNA levels, and MMP2/TIMP2 ratio (E) in control (C), aldosterone-salt-treated animals (ALDO), PASE (PASE), aldosterone-salt plus PASE treated animals (ALDO+PASE), spironolactone treated animals (SPIRO) and aldosterone-salt plus spironolactone (ALDO+SPIRO). Data are expressed as mean ± SEM derived from 8 animals per group. *p<0.05 *vs*. C; ^†^p<0.05 *vs*. ALDO.

HPLC separation of components within PASE employed a method similar to Monagas et al. [43], with a Waters (Milford, MA) Novapak C18 60Å 4 µm, 30×0.39 cm column, pre-equilibrated in Buffer A (2% CH_3_COOH in H_2_O) at a flow rate of 1 ml/min at room temperature. The PASE sample (0.2 g total) was dissolved in buffer A and filtered (0.22 µm) prior to injection, after which a 55 minute linear gradient to 80% buffer B (2% CH_3_COOH, 25% CH_3_CN) was started. The column was then washed with increasing amounts of buffer B as follows (2 min, 80–90% buffer B; 13 min hold at 90% buffer B; 20 min, 90–100% buffer B), with a final 10 min wash with 2% CH_3_COOH, 75% CH_3_CN (total program time = 100 mins). Fractions were collected at 1 minute intervals and the absorbance of the elute monitored at 280 nm, obtaining 100 fractions. To simplify the transactivation assay, pools of every 10 fractions were prepared and labeled P1 to P10 (see Figure 1). Transactivation assays were then performed as described above. The cells were incubated with each pool (P1–P10) and with each corresponding blank to exclude buffer interferences at a dose of 1 µM. Subsequently, transactivation assays were performed using individual fractions of the positive pool which worked as the reference group (aldosterone 1 nM+spironolactone 100 nM).

### Statistical analysis

The data was analysed using a one-way analysis of variance, followed by a Bonferroni test if differences were noted (GraphPad Software Inc., USA). A *p*-value of 0.05 or less was considered significant.

## Results

### Hemodynamic parameters

Table 2 shows hemodynamic values obtained at end of the study. SBP, DBP, LVSP and LVEDP were significantly higher in aldosterone-salt-treated animals than in controls. –dP/dt was lower in aldosterone-salt-treated rats than in controls and +dP/dt was similar in all groups except in the ALDO+PASE group, which was higher than the aldosterone-salt group. PASE treatment prevented the changes observed with aldosterone-salt treatment.

**Table 2:** Hemodynamic parameters and collagen content.

	CONTROL	ALDO	PASE	ALDO+PASE	SPIRO	ALDO+SPIRO
**SBP (mm Hg)**	119±1.9	145±2.4*	114±4.1†	119±2.6†	116±5.4†	113±2.1†
**DBP (mm Hg)**	89±1.2	108±3.1*	91±3.4†	92±4.1†	83±1.2†	91±2.2†
**LVSP (mm Hg)**	121±1.7	146±9.3*	115±2.3†	123±2.5†	117±2.3†	116±1.8†
**LVEDP (mm Hg)**	3.8±0.2	9.7±0.4*	4±0.5†	3.1±0.7†	4.9±1.1†	3.9±0.6†
**HR (BPM)**	385±27	359±46	348±29	391±45	381±38	363±44
**+dP/dt (mm Hg/s)**	9390±163	8177±236	8128±388	10065±416†	8367±325	8230±299
**–dP/dt (mm Hg/s)**	−7106±479	−4859±398*	−6125±356†	−9789±301†	−5988±196†	−6020±241†
**HW/BW (mg/g)**	2.8±0.6	3.1±0.12*	2.6±0.06†	2.7±0.1†	2.4±0.2†	2.5±0.1†
**COLLAGEN (%)**	0.057±0.01	0.220±0.02*	0.065±0.01†	0.051±0.02†	0.042±0.02†	0.065±0.01†

SBP, systolic blood pressure; DBP, diastolic blood pressure; LVEDP, left ventricular end-diastolic pressure; LVSP, left ventricular systolic pressure, HR, heart rate; +dP/dt the first derivative of LV pressure rise over time; –dP/dt, the first derivative of LV pressure decline over time. HW/BW; heart weight/100 g body weight.

*p<0.05 vs CONTROL;

† p<0.05 vs ALDO.

### Hypertrophy and fibrosis

Relative heart weight (HW/BW) was significantly higher in aldosterone-salt-treated rats. These animals showed increased myocardial collagen content (Table 2) as well as increased expression of the genes encoding the fibrotic mediators, transforming growth factor beta (TGF-β) and connective tissue growth factor (CTGF), compared to controls (Figures 1A and 1B). PASE treatment also prevented the increase in these parameters induced by aldosterone-salt treatment. Matrix metalloprotease 2 (MMP2) and matrix metalloprotease inhibitor 2 (TIMP2) mRNA levels were higher but the MMP2/TIMP2 ratio was lower in aldosterone-salt-treated rats compared to controls (Figures 1C, 1D and 1E). PASE treatment also prevented the increase of MMP2 and TIMP2 mRNA levels observed with aldosterone-salt treatment and increased the MMP2/TIMP2 ratio. MMP2/TIMP2 ratio was significantly higher in aldosterone-salt-treated rats when PASE was administered as well as in SPIRO group (Figure 1E).

### Inflammation, oxidation and eNOS

**Figure 2:**
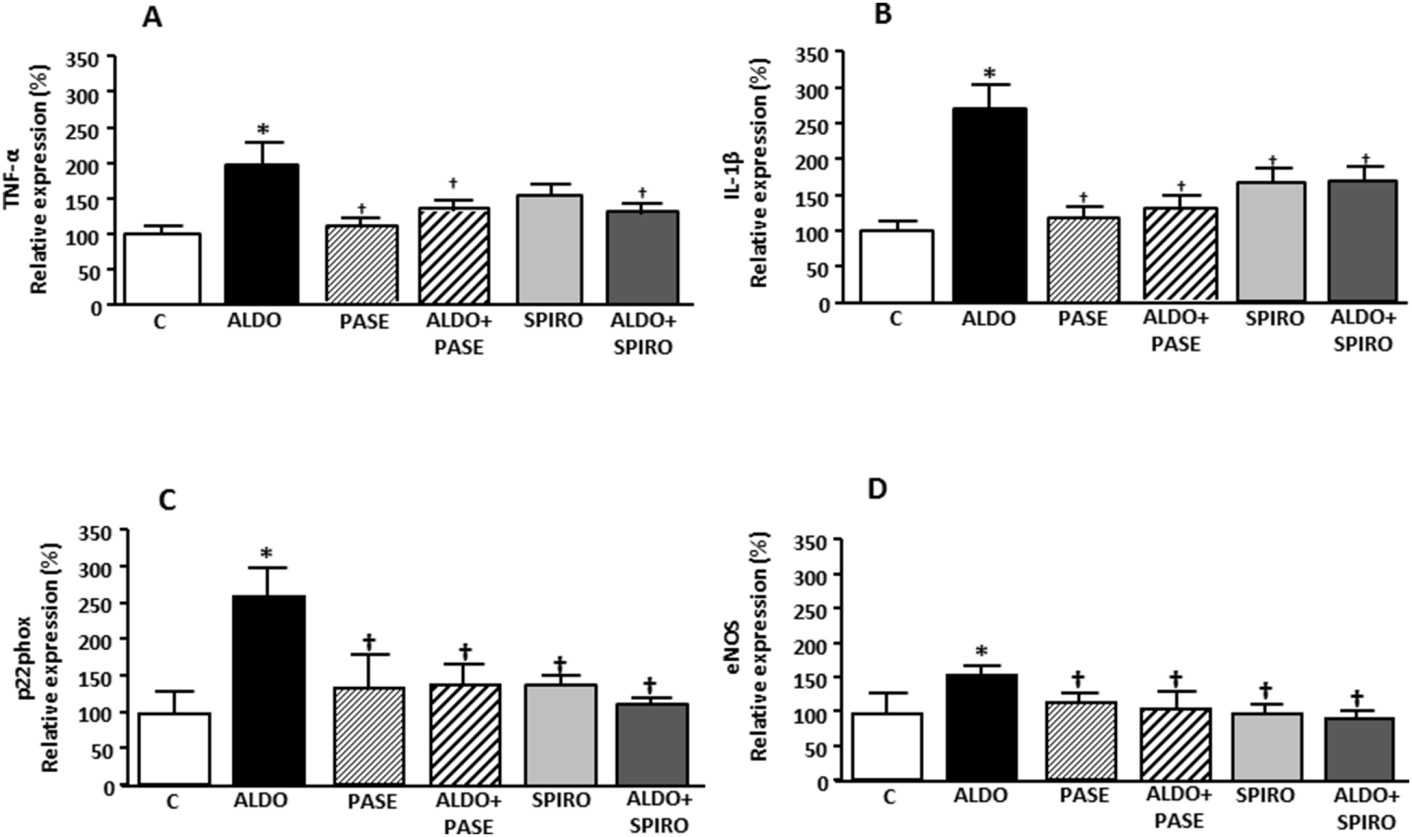
TNF-α (A), IL-1β (B), p22phox (C) and eNOS (D) mRNA levels in control (C), aldosterone-salt-treated animals (ALDO), PASE (PASE), aldosterone-salt plus PASE treated animals (ALDO+PASE), spironolactone treated animals (SPIRO) and aldosterone-salt plus spironolactone (ALDO+SPIRO). Data are expressed as mean ± SEM derived from 8 animals per group. *p<0.05 *vs*. C; ^†^p<0.05 *vs*. ALDO.

Tumor necrosis factor alpha (TNF-α) and interleukin 1 beta (IL-1β) mRNA levels were higher in aldosterone-salt-treated rats than in controls; these levels were reduced in ALDO+PASE and ALDO+SPIRO groups (Figures 2A and 2B) compared to aldosterone-salt treated rats. Similarly, aldosterone-salt-treated rats showed increased p22phox and endothelial nitric oxide synthase (eNOS) mRNA levels compared to control rats. Treatment with PASE when administered with the aldosterone-salt decreased the levels of both oxidative parameters compared to aldosterone-salt-treated rats (Figures 2C and 2D).

### SGK-1 expression

**Figure 3:**
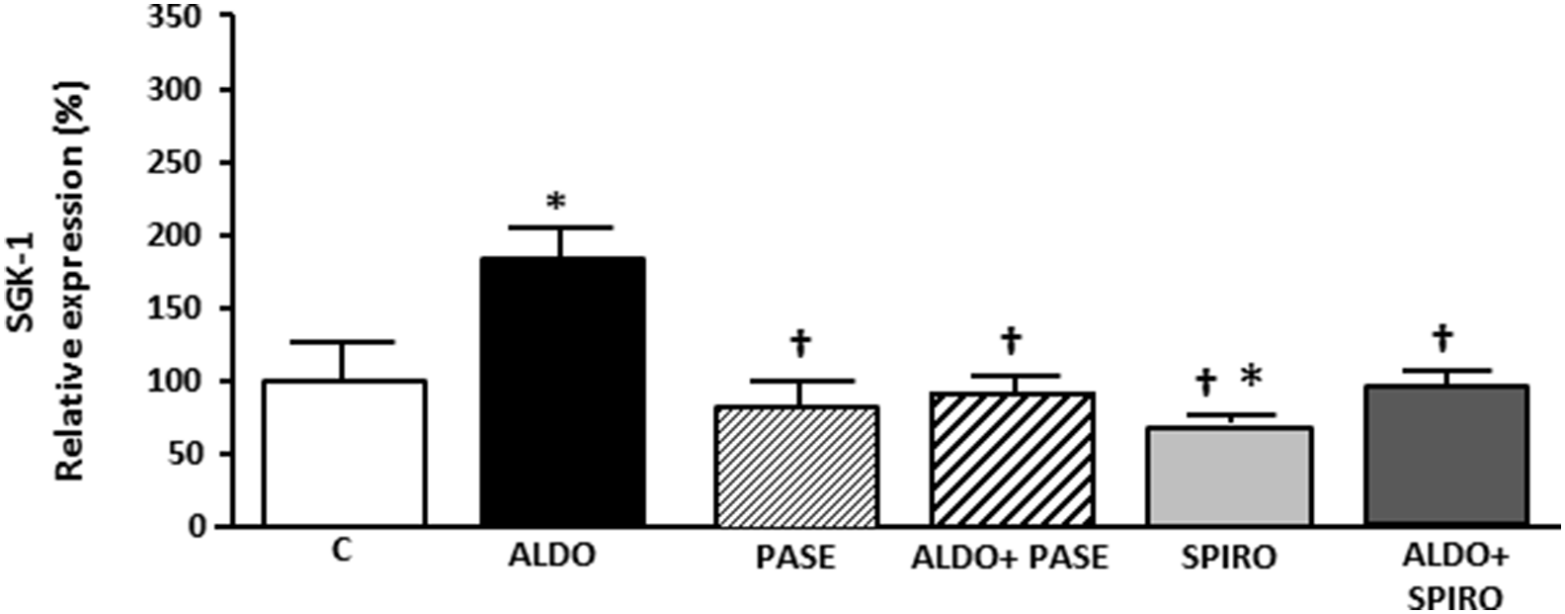
SGK-1 mRNA levels in control (C), aldosterone treated animals (ALDO), PASE (PASE), aldosterone plus PASE treated animals (ALDO+PASE), spironolactone treated animals (SPIRO) and aldosterone-salt plus spironolactone (ALDO+SPIRO). Data are expressed as mean ± SEM derived from 8 animals per group. *p<0.05 *vs.* CONTROL; ^†^p<0.05 *vs.* ALDO.

All of the aforementioned changes induced by aldosterone-salt were accompanied by increased SGK-1 mRNA levels, which were markedly reduced when aldosterone-salt rats were treated with PASE (Figure 3).

### Proanthocyanidins identification

**Figure 4:**
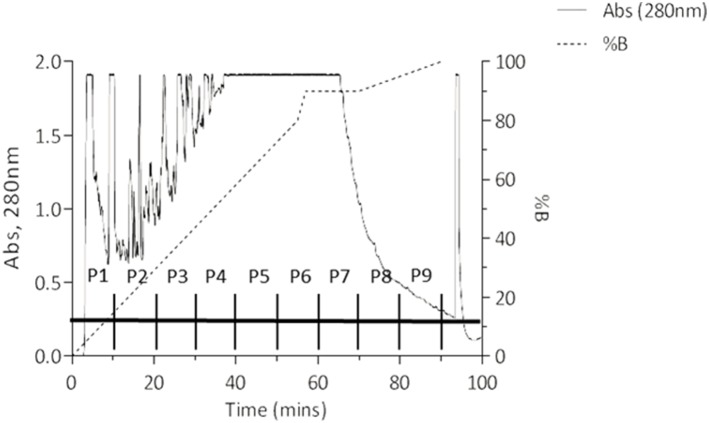
HPLC chromatogram and pools representation. Column: Waters Novapak C18 60Å 4 µm, 30×0.39 cm. Flow rate = 1 ml/min. Buffer A: 2% CH3COOH. Buffer B: 2% CH3COOH, 25% CH3CN. Arrow indicates column strip with 2% CH3COOH, 75% CH3CN. Sample volume loaded = 1 ml. Fractions collected at 1 min intervals. Pools compound of 10 individual fractions; P1∶1–10, P2∶11–20, P3∶21–30. P4∶31–40, P5∶41–50, P6∶51–60, P7∶61–70, P8∶71–80, P9∶81–90 and P10∶91–100.

Fractions were collected at 1 minute intervals and the absorbance of the elute monitored at 280 nm, which yielded 100 fractions. Proanthocyanidin were identified from the chromatogram obtained by monitoring the absorbance and based on Monagas et al [43] where they identified proanthocyanidins between 20 and 40 minutes after sample was injected in the HPLC (Figure 4).

### Transcriptional activity of mineralocorticoid receptor

**Figure 5:**
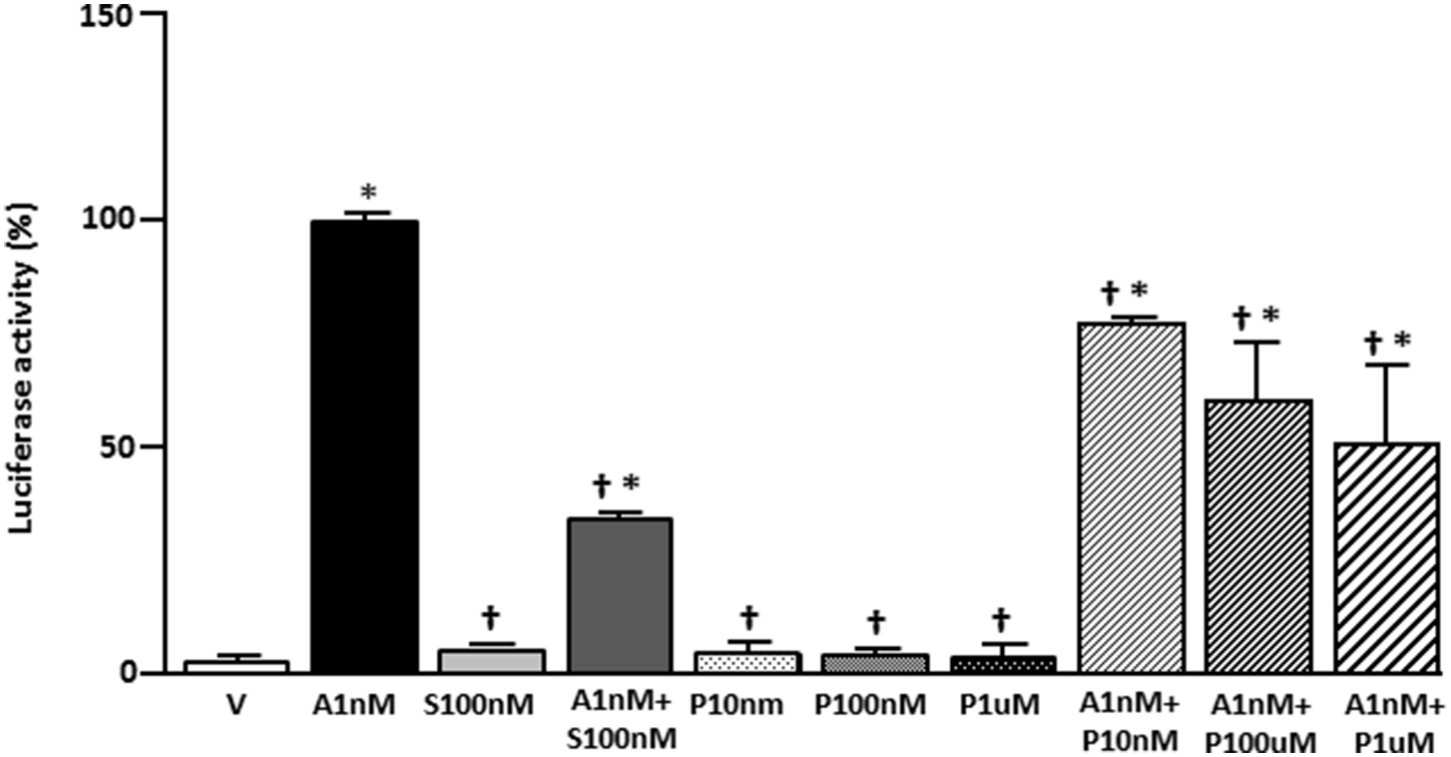
Effect of PASE on aldosterone-induced human mineralocorticoid receptor (hMR) transcriptional activity. CV-1 cells transfected with hMR expression and MMTV-LUC reporter plasmids were treated with vehicle (V) or aldosterone 1 nM (A1 nM) in presence of spironolactone 100 nM (S100 nM) or PASE 10 nM, 100 nM or 1 µM (P10 nM, P100 nM or P1 µM). Each data point represents the mean ± SEM derived from 3 independent experiments. *p<0.01 vs V; ^†^p<0.05 *vs.* A1 nM.

To assess whether the results obtained with PASE treatment, which paralleled those with spironolactone, represent specific antagonism of the MR, the ability of the total extract to block MR activity in a transcriptional assay was examined. PASE treatment was without activity alone and resulted in an antagonism of the response to aldosterone (Figure 5).

**Figure 6:**
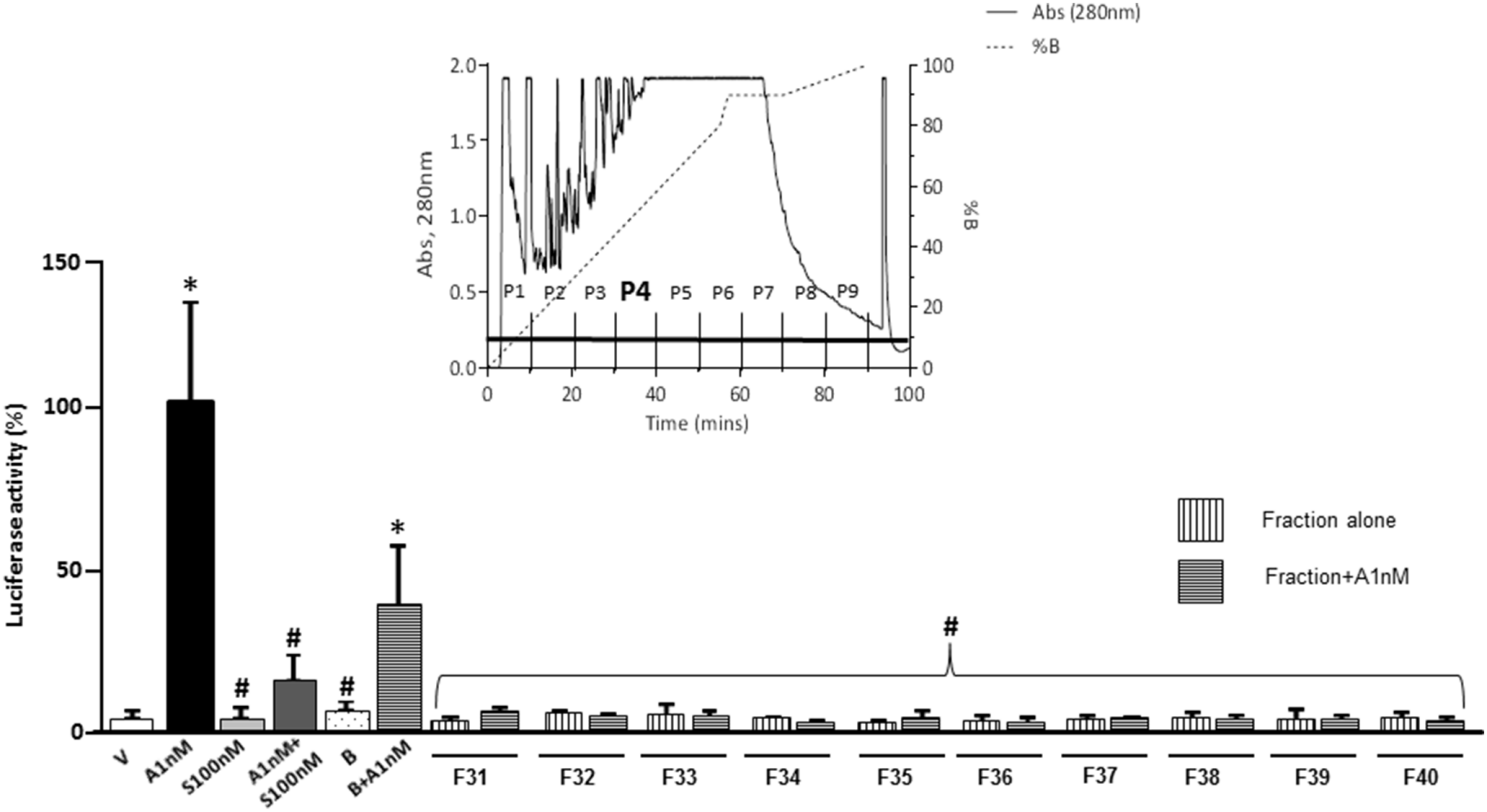
Effect of P4 individual fractions on aldosterone-induced human mineralocorticoid receptor (hMR) transcriptional activity. CV-1 cells transfected with hMR expression and MMTV-LUC reporter plasmids were treated with vehicle (V) or aldosterone 1 nM (A1 nM) in presence of spironolactone 100 nM (S100 nM) or fractions (F) 31 to 40 (1 µM), blank (B) and blank+aldosterone1 nM (B+A1 nM). Each data point represents the mean ± SEM derived from 3 independent experiments. *p<0.01 vs V; ^†^p<0.05 *vs.* A1 nM.

Aldosterone-induced MR transactivation was assessed in the presence of the PASE fractions obtained by HPLC. We observed a decrease of MR activity in pooled fractions from P2, P4, P5, P7 and P10 (data not shown). The ability of each of these fractions alone to inhibit the aldosterone-induced MR transactivation was examined. P4 and P10 were able to block the MR mediated response to aldosterone (Figure 6). Since Fractions in P10 are at the end of the column when high CH_3_CN was used to strip the column, we did not investigate these further. We therefore assessed the MR activity of the individual fractions (F31 to F40) which formed the P4 pool. Individual fractions from F31 to F40 were able to block MR activity (Figure 6).

## Discussion

The present study shows for the first time that the treatment with a proanthocyanidins-rich almond skin extract prevents cardiac hypertrophy, fibrosis, inflammation, oxidative stress, hypertension and diastolic dysfunction induced by aldosterone plus salt administration in rats. PASE is able to reduce gene expression of the aldosterone-induced gene SGK-1 too. Furthermore, the study identifies proanthocyanidins as the PASE compounds responsible for the beneficial cardiac effects observed reducing aldosterone-induced transcriptional activity of the mineralocorticoid receptor *in vitro*. The effects of PASE treatment in aldosterone-salt rats on cardiac parameters were paralleled to those observed with the mineralocorticoid receptor antagonist spironolactone.

Many studies have explored the beneficial effects of proanthocyanidins in a purely descriptive way focusing on putative antioxidant effects. In the present study, we provide additional original, mechanistic insight into the effect of this extract on cardiac pathophysiology.

A large number of studies have described, in both *in vitro* and *in vivo* models, the numerous effects of proanthocyanidins-rich extracts on cell signaling. Although these studies are of significant value as a starting point to define the health benefits of proanthocyanidins, they have very limited value when mechanisms of action are discussed, given the difficulty in identifying the molecule(s) responsible for the observed effects. In the present study we have been able to describe not only the mechanism through which PASE is able to induce beneficial cardiac effects but also the molecules responsible for the observed effects. PASE is able to decrease the transcriptional activity of the mineralocorticoid receptor as was seen with the mineralocorticoid receptor antagonist, spironolactone. This result is particularly interesting since we are unaware of previous studies of a rich proanthocyanidins extract acting as a steroid receptor antagonist. Moreover, the HPLC results corroborated that proanthocyanidins are the phenolic compounds of PASE responsible of cardiac beneficial effects observed in aldosterone-salt-treated rats. Previous studies have shown that phenolic compounds could interact with cell signaling pathways. In animal models, it has been shown that dietary polyphenols can modulate expression of numerous genes and proteins in different organs, such as the aorta of apolipoprotein E-deficient mice [44]. This capacity of polyphenols to modulate the expression of genes through modulation of cell signaling pathways has also been described in vitro, e.g. epigallocatechin gallate in hepatocytes [45], flavonoids in vascular endothelial cells [46], [47], or neuronal cells [48]. Recent studies have also shown that polyphenols can modulate other regulators involved in the posttranscriptional regulation of expression of genes, particularly microRNA [49]. Nevertheless, none of these studies have revealed before an improvement of cardiac function related to mineralocorticoid receptors and pointed out proanthocyanidins as the main phenolic compound responsible of the beneficial effects observed in the present study. The study shows also for the first time that PASE was able to reduce the elevated mRNA levels of SGK-1 in aldosterone-treated rats. We have shown that SGK-1 is overexpressed in the heart of aldosterone+salt treated rats were structural, functional and molecular cardiac alterations occur [25]. This allows us to postulate that this mechanism has a central role in the amelioration and normalization of the complex intracellular signaling, involving fibrotic, inflammatory and oxidative pathways, which led to the cardiac hypertrophy and fibrosis induced by aldosterone.

Anti-inflammatory, antioxidant and antifibrotic effects shown in the present study, contribute to the beneficial cardioprotective effects of PASE in aldosterone-salt-treated rats. PASE reduced elevated TNF-α and IL-1β mRNA levels in hearts from aldosterone-salt-treated rats. Previous studies have reported that regular almond proanthocyanidins intake reduce inflammatory biomarkers [50]–[52]. Cell culture experiments, animal studies and human intervention trials have already shown that flavonols and procyanidins exhibit anti-inflammatory and antioxidant properties acting via several molecular targets, involving NF-κB and iNOS regulation [53]. Consumption of grape seed proanthocyanidins reduced levels of hs-C-reactive protein, interleukin-6 (IL-6) and TNF-α in Zucker *Fa/fa* rats fed a hyperlipidemic diet [54]. In addition to its anti-inflammatory effect, PASE reduced elevated p22phox mRNA levels in aldosterone-salt-treated rats, indicating a potential reduction of oxidative stress. Chronic ingestion of anthocyanins was associated with increased cardiac glutathione concentrations in rats [55]. Moreover, green tea epigallocatechin gallate reduced oxidative stress and bcl-2 levels in hypertrophied rat hearts [56].

Left ventricular remodeling is regulated in an important extent by eNOS and NO signaling [57]. Mice lacking eNOS display significantly aggravated left ventricular remodeling after myocardial infarction compared to wildtypes [58]. Furthermore, left ventricular function was improved, and hypertrophy was reduced in animals with selective overexpression of eNOS in cardiomyocytes [59]. Elevated eNOS expression in aldosterone-salt-treated rats could be considered as a defense mechanism against cardiac hypertrophy and elevated blood pressure, leading to enhance NO release which would be offering cardiac protection [60]–[62]. This concept is further supported by the fact that the reduction of p22phox expression by PASE, and potential reduction of oxidative stress, was accompanied by reduction of eNOS expression.

Aldosterone-salt treatment increased relative heart weight and collagen content reflecting an increase in collagen synthesis by fibroblasts as a reparative response to inflammation and cell death, and to hypertrophy of myocytes [63]–[65]. Our results have shown a decrease in the relative heart weight and collagen content with PASE treatment. Several studies have shown that polyphenols are cardioprotective by reducing cardiac hypertrophy. Administration of an alcohol-free red wine in rats with postinfarction remodeling showed a protective effect on hearts by repressing hypertrophy-associated phosphorylation of protein kinase C (PKC) α/β II and by activating Akt/protein kinase B [66]. PASE treatment reduced overexpression of fibrotic mediators suggesting a novel beneficial effect of proanthocyanidins on cardiac remodeling. Decreased production of MMPs or stimulation of TIMPs will also contribute to fibrosis. Both MMP2 and TIMP2 were elevated in aldosterone-salt-treated rats compared to controls; elevation of TIMP2 was more marked than that of MMP2, and as a consequence, the MMP2/TIMP2 ratio was reduced. These parameters were normalized by PASE, consistent with the ability of PASE to decrease the increased cardiac collagen content and fibrosis induced by aldosterone-salt-treatment.

The beneficial effects of PASE on cardiac hypertrophy, as well as on inflammatory, oxidant and fibrotic mediators, could contribute to the amelioration of cardiac hemodynamic parameters in aldosterone-treated rats. In fact, treatment with PASE reduced SBP and DBP, normalized LVSP and LVEDP, increased +dP/dt and –dP/dt, indicating an improvement of diastolic dysfunction and a stimulation of contractile capacity. PASE also showed an antihypertensive action in aldosterone-treated rats. Previous studies have already reported beneficial effects of flavonoids on blood pressure. Ingestion of green tea containing catechins for 24 weeks, reduced systolic blood pressure, in obese or pre-obese Japanese children [67], and epigallocatechin gallate reduced diastolic blood pressure in overweight or obese adults [68].

Taken together, the beneficial cardiac effects of PASE on the structural, functional and molecular alterations induced by the administration of aldosterone, our results suggest that one of the mechanism through which PASE proanthocyanidins could be acting would be as a mineralocorticoid receptor antagonist. The hypothesis is supported by the reduced expression of the principal mediator of cellular actions of aldosterone, SGK-1, to a similar extent to that observed with MR antagonist together with the decreased aldosterone-induced transactivation of the MR induced by PASE treatment. Moreover, we identified proanthocyanidins as the PASE compounds able to interact with MR and decrease aldosterone-induced transactivation of the MR. Our study provides a novel insight into mechanisms, other than an antioxidant action of proanthocyanidin-rich compounds that mediate their beneficial effects in a model of cardiovascular disease. However, further studies are needed to confirm this hypothesis.

In conclusion, the effects of PASE on cardiac hypertrophy, fibrosis, hypertension and diastolic dysfunction, were associated with a reduction of inflammatory, oxidative and fibrotic mediators. The observed reduction of SGK-1 expression together with the ability of PASE proanthocyanidins to antagonize MR-mediated transactivation *in vitro* argues for a MR-antagonist effect of this proanthocyanidins-rich extract.
